# Intraspecific differences in *Androctunus crassicauda* venom and envenomation symptoms

**DOI:** 10.17179/excli2022-5078

**Published:** 2022-09-21

**Authors:** Mehdi Safdarian, Babak Vazirianzadeh, Ahmad Ghorbani, Narges Pashmforoosh, Masoumeh Baradaran

**Affiliations:** 1 Nanotechnology Research Center, Ahvaz Jundishapur University of Medical Sciences, Ahvaz, Iran; 2 Social Determinant of Health Research Center, Ahvaz Jundishapur University of Medical Sciences, Ahvaz, Iran; 3 Toxicology Research Center, Medical Basic Sciences Research Institute, Ahvaz Jundishapur University of Medical Sciences, Ahvaz, Iran

**Keywords:** Androctunus crassicauda, intraspecific differences, envenomation symptoms, venom difference

## Abstract

Envenomation by *Androctunus crassicauda* is very frequent in Iran, especially in the south-west*.* This scorpion is one of the six scorpions whose venom is used to prepare anti-venom. Using HPLC, we discovered venom components of *A. crassicauda* varies from one specimen to another depending on geographical location, and this result is confirmed by those first found in various symptoms of *A. crassicauda* sting in envenomed persons from two separate geographical places (north and south of Khuzestan province). There was a significant relationship between symptoms and location of envenomation by *A. crassicauda*. Muscle spasm was more dominant in envenomed people from Northern cities, and venom chromatogram analysis showed the presence of at least six main sharp peaks in Northern *A. crassicauda* rather than Southern* A. crassicauda*. It shows intraspecific differences in venom of *A. crassicauda* that must be considered in treatment of stung people from different geographical locations as well as in the preparation of anti-venom.

See also Figure 1(Fig. 1).

## Introduction

Scorpion envenomation is a global health problem in tropical and subtropical regions of the world. Iran is a high-risk region in regard to scorpion stings (Dehghani et al., 2018[6]; Rafizadeh et al., 2013[29]; Nejati et al., 2018[24]; Ghorbani et al., 2021[17]). According to the statistics released by the CDC of the Iranian Ministry of Health and Medical Education, Iran has the second rank of venomous animal stings in the world by a recording of about 250,000 stings, following Mexico (Firoozfar et al., 2019[15]). According to the last reports by 2017, there are 64 scorpions, belonging to 23 genera in Iran which are classified into three families: Buthidae (86 %), Scorpionidae (9.5 %), and Hemiscorpiidae (4.5 %) (Dehghani and Kassiri, 2018[8]). Scorpions of the Buthidae family are responsible for about 86 % of the documented cases of scorpion stings in Iran (Firoozfar et al., 2019[15]). In this country, 12 species of eight genera are classified as medically important scorpions: *Hemiscorpius, Androctonus*, *Odontobuthus*, *Apistobuthus, Compsobuthus*, *Hottentotta*, *Orthochirus*, and *Mesobuthus *(Sanaei-Zadeh et al., 2017[30]), which except *Hemiscorpius*, the rest belongs to Buthidea family. Among them,* Androctonus crassicauda* is the most dangerous species of the Buthidea family. This species' mortality was documented in Khuzestan, North West Iran, East Azerbaijan, and West Azerbaijan. In the Middle East, it is also known as the medically important scorpion species (Firoozfar et al., 2019[15]). Khuzestan has the highest rate of scorpion stings in Iran based on reporting annually scorpion stings, about 541 cases per hundred thousand people (Rafizadeh et al., 2013[29]). Climate factors and habitat type are the most important determinants of the development of these venomous animals in Khuzestan (Ghorbani et al., 2021[17]). *A. crassicauda, Hemiscorpius lepturus, and Mesobuthus eupeus* are responsible for most of the reported scorpion sting cases in this province. After *H. lepturus, A. crassicauda *is the second most common cause of scorpion sting in south-west Iran (Radmanesh, 1990[28])*.*

*A. crassicauda *that is also known as the black scorpion varies from brown to black, in color. It lives commonly under the rocks, in sand dunes, and the crevices of the earth (Dehghani and Fathi, 2012[7]). Due to stimulating the acetylcholine receptors through the human body, the venom of *A. crassicauda* can cause autonomic, CNS, and muscle function disturbances (Radmanesh, 1990[28]; Sanaei-Zadeh et al., 2017[30]). Local pain, which may spread to regional lymph nodes, hyperemia, and edema, is the characteristic clinical sign of *A. crassicauda* sting (Garcia et al., 1999[16]). Local muscular spasms and widespread muscle paralysis are the most prevalent systemic symptoms (Radmanesh, 1990[28]). Chemical and biological analysis of the scorpion venom revealed some peptides and proteins which are responsible for the sting symptoms (Delgado-Prudencio et al., 2022[9]; Petricevich, 2010[27]). High-performance liquid chromatography (HPLC) is one of the suitable instrumental methods to obtain the protein pattern of scorpion venom (Batista et al., 2007[2]; Tahir et al., 2021[33]). 

In the symptom of patients stung by *A. crassicauda* referred to the Toxicology Unit of Ahvaz Razi Hospital (Khuzestan province), some obvious differences were observed in patients from northern and southern regions of the province. As a result, we evaluated the differences between *A. crassicauda* scorpion stings in the northern and southern towns of Khuzestan province, as well as the association between the geographical location of envenomation and the severity of the patients' problems. Moreover, the chromatographic profiles of *A. crassicauda* venom were conducted on reversed-phase high-performance liquid chromatography (RP-HPLC) to compare the protein pattern of the two geographically-separated *A. crassicauda *venoms and report the relationship between these protein patterns and clinical observations.

## Materials and Methods

### Clinical observation, documentation and statistical analysis

This study was conducted in the Clinical Toxicology Unit of Ahvaz Razi Hospital (Khuzestan province, Iran), the reference hospital in the southwest of the country for scorpion stings, from March 21, 2020, to January 10, 2021. After obtaining ethics approvals from the Research Ethics Committee (approval number: IR.AJUMS. REC.1398.818 on Nov. 26, 2020) and written informed consent, people who were envenomed by *A. crassicauda* and referred to Ahvaz Razi Hospital were selected for the study. Age, sex, patient residency (North or South of Khuzestan), stung location (upper limb, lower limb, head and neck, trunk), and sting symptoms were all recorded in the medical records. Patients who were sent to Razi Hospital and brought the scorpion that stung them with them entered the research when the scorpion species was confirmed (*A. crassicauda*). The scorpion species were determined according to Farzanpay's key of identification (Farzanpay, 1988[14]).

The included envenomed people were classified into two categories based on the patient residency: 1) envenomed in northern cities of Khuzestan (in brief, EiNK) including, Andimeshk, Lali, Gotvand, Izeh, and Bagh-malek; and 2) envenomed in southern cities of Khuzestan (in brief, EiSK) including, Abadan, Khoramshahr, Ramshir, Mahshahr, Hendijn, Shadegan, and Behbahan. Envenomation symptoms were classified into three categories: 1- severe pain, 2- mild pain, and 3- muscle spasm with pain. Treatment was performed with repeated IV injections of calcium until relief of spasm and pain symptoms (1 to 5 doses, 10 mg of calcium per dose). The duration of hospitalization was based on the severity of scorpion symptoms, which was divided into 4 categories: 6-12 hours, 12-24 hours, 24-48 hours, and more than 48 hours.

Finally, statistical relationships were determined between patient residency (north or south of the Khuzestan) and following variables: sting symptoms, number doses of calcium prescribed, and duration of hospitalization. The data were compiled using Microsoft Excel^® ^and analyzed using the software SPSS^®^. The *mean*±*SD *of the variables was calculated. Chi-square test in the contingency table was also used to evaluate the hypothesis of the relationship between the area of a sting by *A. crassicauda* and other variables considered. In cases in which the expected number of observations in the cells of the table was less than 5, Fisher's exact test was used.

### RP-HPLC analysis of A. crassicauda venom 

#### Venom preparation

*A. crassicauda *were hunted from north and south of Khuzestan province and were kept in two separate containers until milking. Venoms of scorpions were obtained by electroshock method and were freeze-dried. Crude venom (5 mg) from *A. crassicauda* of the north (NAc) and *A. crassicauda* of the south (SAc) was diluted in 0.5 ml deionized water and centrifuged at 6000 rpm for 17 minutes to remove insoluble material. The research used the supernatant, which included the solubilized venom.

#### RP-HPLC pattern of crude scorpion venoms

RP-HPLC was performed on Agilent 1260 infinity (II) system (Agilent Technologies, Santa Clara, CA) equipped with a photodiode array detector (PDA) comprising an in-line degasser, and a high-pressure manual Rheodyne injection valve (20 µL injection loop). The sample components were separated using a C18 reversed phase column Zorbax 300SB‐C3 (4.6 × 150 mm, particle size 5 μm; Agilent Technologies) equipped with a 1 cm guard column having the same stationary phase as the analytical column. The mobile phase is composed of (A) 0.1 % trifluoroacetic acid, and (B) acetonitrile. Binary gradient elution profile of the mobile phase was: t = 0, 100 % A; t = 60, 100 % B. The mobile phase flow rate was 0.5 mL min^−1^. The peaks were monitored by absorption at 280 nm.

## Results

A total of 98 patients, 50 men and 48 women stung by *A. crassicauda*, were included in the study. The mean age of participants was 35.5 years (range: 18-90 years). Among them, 44 cases (44.9 %) were EiNK and 54 patients (55.1 %) were EiSK (Table 1(Tab. 1)). The sting site was the upper extremity in most of the cases (66, 67.3 %), while 32 cases (32.7 %) were stung at the lower extremity. Sting in the trunk and head & neck was not seen in any of the patients included in the current study. Thirty-nine cases (39.8 %) had mild pain, 49 (50 %) cases showed severe pain, and 10 (10.2 %) presented muscle spasms with pain. Just 2 cases (20 %) of spasm with pain patients were EiSK and the other (80 %) were EiNK.

In total, 13 cases did not need to receive calcium, which among them 12 patients (92.3 %) were EiSK, and 1 (7.7 %) was EiNK. Among EiNKs, 3 cases (3.1 %) received 4 doses of calcium and 3 cases (3.1 %) received 5 doses of calcium. It was while there was no evidence of the need for 4 or 5 doses of calcium in EiSKs. Overall, 70.4 % of patients were hospitalized for 6-12 hrs or 12-24 hrs, of which 47.9 % were EiSK and 23.45 % were EiNK. Conversely, the number of 24-48 or more than 48-hour hospitalization was more related to EiNKs as follows: 21.4 % and 8.2 % of 24-48 hrs or more than 48 hrs hospitalization were related to EiNK and EiSK, respectively.

**Figure 2:**
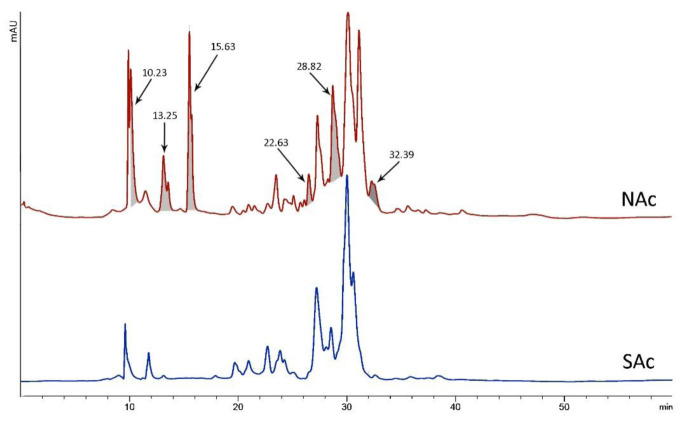
HPLC chromatograms for *A. crassicauda* venom living in northern Khuzestan (NAc) and *A. crassicauda *living in southern Khuzestan (SAc). The main differences between the two chromatograms are highlighted.

Chi-square and Fisher's exact tests were used to analyze the connection between patient residence location and other factors. Table 2(Tab. 2) shows the findings along with the P-values obtained. A statistically significant difference was found between the residency of patients and 3 variables including: sting signs, number dose of needed calcium, and duration of hospitalization (p < 0.05). The dissolved crude venom was passed through a C18 reverse-phase column and their chromatograms were compared to analyze the differences in the venom components of *A. crassicauda* species inhabiting in the north and south of Khuzestan province. Following separation by HPLC, typical chromatographic profiles for both venoms, SAc and NAc, exhibit the similar pattern, as illustrated in Figure 2(Fig. 2). However, the NAc venom profile fluctuated from the SAc venom chromatogram at six main sharp peaks with retention time (RT) of 10.23, 13.25, 15.63, 22.63, 28.82, and 32.39 min (marked in Figure 2(Fig. 2)). Although it is not possible to accurately identify the type of peptides or molecular weight of the components in these chromatograms, it is clear that there are at least six proteins or peptides in NAc venom that are not present in SAc venom.

The different main peaks between NAc and SAc venom chromatograms can be divided into two categories: peaks locating below 20 min (10.23, 13.25, and 15.63 min); and peaks located at 22.63, 28.82, and 32.39 min (Figure 2(Fig. 2)). Due to using a C18 reverse-phase column, the peaks seen in the less RT were the components that have more polarity. Thus, components with retention time below 20 min have more polarity than those located after 20 min. In a further study, each peptide could be identified and purified by collecting the fractions of each chromatogram with improving methods.

## Discussion

*A. crassicauda* is one of the highly toxic scorpions (Bonnet, 1997[3]), and one of the most important scorpions existing in Iran (Jalali and Rahim, 2014[18]). Geographical variations in venom composition were studied in several scorpion species (Mathe-Hubert et al., 2019[21]), but surprisingly never in *A. crassicauda*. The current research not only provides intriguing data regarding differences in geographically distant *A. crassicauda* venom for the first time, but it also includes a statistically detailed description of clinical data that has not been done in prior variation studies.

According to the evidence collected and analyzed from 98 *A. crassicauda*-envenomed people referred to Ahvaz Razi Hospital, there is a statistically significant relationship between the geographical location and symptoms of a sting by *A. crassicauda* (particularly muscle spasm), length of hospitalization, and the amount of calcium intake by patients. Muscle spasms and severe pain were more seen in people envenomed by NAc rather than people envenomed by SAc. Hospitalization and calcium dosage requirements were higher in NAc-envenomed patients. HPLC study of *A. crassicauda* specimens taken from the north and south of Khuzestan province revealed that venom composition differs depending on the geographic origin of the species.

Variations in the venom of the same scorpion species in different geographical regions was previously reported in some scorpion species (Amaral and Rezende, 2000[1]; Bücherl, 1971[4]). El Ayeb and Rochat determined the concentration of three neurotoxins (toxins I, II, and III) in the venom of the scorpion *Androctonus australis* Hector using highly specific radioimmunoassays. They found variations of these toxins from individuals collected from different geographic origins (El Ayeb and Rochat, 1985[11]). Similar results were achieved by Martin et al., who reported polymorphism in protein contents of individual *Androctonus australis* Hector venom by the RP-HPLC chromatograms of three individual venoms of this scorpion. Even though extracts show homogeneous fractions, considerable quantitative variation is predicted in non-toxin venom (Martin et al., 1987[20]). A varied range of protein contents were obtained in several venom extractions from different cases of the *Androctonus mauretanicus mauretanicus *scorpion, and the results show that venom lethality varies from specimen to specimen and those pharmacokinetic parameters of venom and anti-venom are totally different (El Hafny et al., 2002[12]). The intraspecific variation in the venom of four populations of *Parabuthus granulatus* collected from geographically diverse areas in southern Africa was recently discovered using hierarchical clustering analysis (Schaffrath et al., 2018[31]). 

Similar to previous studies, in the current study, HPLC analysis showed venom components of *A. crassicauda* vary from specimen to specimen with the geographic origin of the scorpion. NAc venom differs from SAc venom in at least six components, three of which are more polar. These results were expected because a difference was also seen in the symptoms of *A. crassicauda*-envenomed people in north and south of Khuzestan which could be explained by differences in the venom gland contents.

Even some studies pointed out that venom composition and toxicity can be changed in individual scorpion specimens from the same geographic region. Ozkan and Ciftci showed considerable variation in crude venom of different individual *Mesobuthus gibbosus* in the same geographic region in Turkey, based on the electrophoretic profile (Ozkan and Ciftci, 2010[26]). Equivalent results were reported in the venom of the scorpion *Tityus* *serrulatus *of Brazil (enzyme-linked immunosorbent assay (ELISA))*,* whose venom composition varied between several specimens, and the lethality levels changed in the same scorpion species collected from the same zone.

El Ayeb and Rochat (1985[11]) assign an important role of ecological factors to the variations in toxin content of *Androctonus australis*. The geographic origin appears to play a role in the variation of *A. crassicauda* venom. Studies have already shown that genetic changes result from a change in the environment, which is defined as a plastic response (Noble et al., 2019[25]). As a result, location-dependent venom component variation might constitute a plastic response to environmental change. It was proposed that scorpions change and improve their venom components in order to adapt to various environments (Evans et al., 2019[13]). 

Although many studies revealed that geographical differences lead to venom differences in scorpions, some previous studies have shown an association between sex (Miller et al., 2016[23]; Schwartz et al., 2008[32]) and size (McElroy et al., 2017[22]) of scorpions with venom differences. Miller et al. suggested that there are intersexual quantitive differences in the venom of *Centruroides vittatus* scorpion (Miller et al., 2016[23]), while a qualitative sex-related difference was seen in the venom of *Opisthacanthus cayaporum *venom (Schwartz et al., 2008[32]). Characterizing the venom expression of the mature and immature size of *Centruroides vittatus *revealed an ontogenetic difference in the venom of this scorpion.

The above-explained study on the venom of *Tityus* *serrulatus* attributed that the variation in the protein contents and expression levels of toxin types could reflect the different symptoms in the victims of scorpion envenomation (Kalapothakis and Chávez-Olórtegui, 1997[19]) similar to what was seen in the envenomed people by *A. crassicauda*. Muscle spasms and severe pain were more seen in referents from the northern location of Khuzestan, where the *A. crassicauda* collected from there showed more peaks in the HPLC chromatogram. As a result, these peaks are thought to be chemicals that have produced more severe symptoms in patients. Despite the fact that the kind of these substances is unknown, the findings are curiously compatible with clinical situations.

According to previous studies, the venom of *A. crassicauda* increases the release of acetylcholine at the neuromuscular junction by both increasing quantal content and the release after single shock stimulation (Vatanpour, 2003[34]; Edwards et al., 1999[10]). Since acetylcholine triggers muscle contraction (Cetin et al., 2020[5]), the muscle spasms observed in the current study in people poisoned by *A. crassicauda* in northern Khuzestan may be due to the activity of one or more components of this scorpion's venom responsible for the increase in acetylcholine release. Considering the chromatogram of NAc venom showed at least six peaks more than SAc venom, it seems these additional peaks are related to compounds whose function is to increase acetylcholine release.

## Conclusion

In conclusion, the venom components of *A. crassicauda* vary dependent on the geographical region that could result in different symptoms in people who are stung by this scorpion. These differences seem to be mostly connected to peptides or proteins that work to increase acetylcholine release. This location-dependent variation in *A. crassicauda* venom necessitates specific consideration in both the treatment of patients envenomed in various places and the manufacturing of anti-venom.

## Declaration

### Acknowledgment

The authors would like to express their gratitude to Ahvaz Jundishapur University of Medical Sciences for financial support (grant number: TRC9906).

### Conflict of interest

The authors declare that they have no conflict of interest.

### Statements and declarations

The authors declare that they have no known competing financial interests or personal relationships that could have appeared to influence the work reported in this paper.

### Ethical statement

Ethics approval was obtained from the research Ethics Committee (approval number: IR.AJUMS. REC. 1398.818 on Nov. 26, 2020) and all data from patients were collected after written informed consent.

## Figures and Tables

**Table 1:**
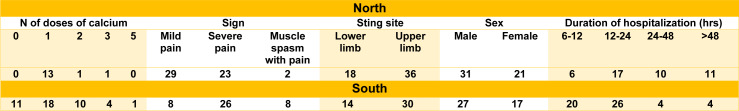
Data from stung people by *A. crassicauda* referred to Razi Hospital

**Table 2:**
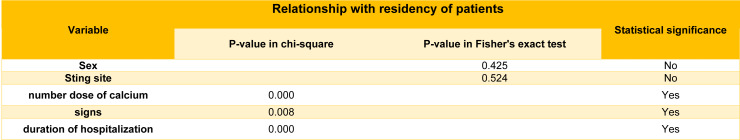
Relationship between residency of patients and five variables

**Figure 1:**
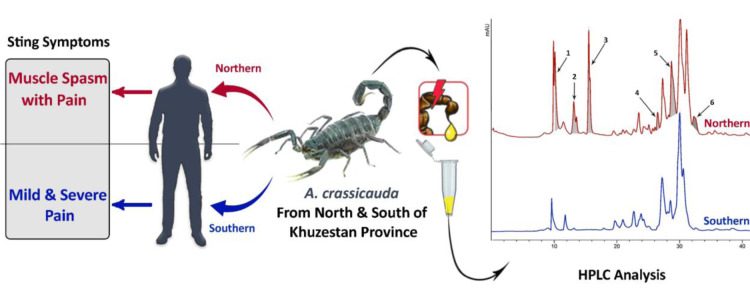
Figure 1: Graphical abstract
